# Amyloid-based therapies did fail again! It is the right time to change our vision on building block of Alzheimer's disease

**Published:** 2014

**Authors:** Morteza Mahmoudi

**Affiliations:** Department of Nanotechnology AND Nanotechnology Research Center, School of Pharmacy, Tehran University of Medical Sciences, Tehran, Iran

**Keywords:** Amyloid, Alzheimer's Disease, Therapy, Dementia

Alois Alzheimer, the Bavarian neuropathologist, first introduced the Alzheimer's disease (AD) in 1906 following detection of amyloid plaques, neurofibrillary tangles, and arteriosclerotic changes in the brain of his patient. AD is known as the most frequent global form of dementia, these days, representing 50-80% of all cases^1^ with huge patient maintenance costs (i.e. 172 billion US dollars in 2010 at the United States^1^ predicting to reach 1 trillion dollars by 2050).^2^ The most well recognized hallmarks of AD neuropathology known now are the extracellular amyloid plaque burden, intracellular neurofibrillary tangles, and microtubule destabilizations.^3^ Misfolding each of these proteinaceous entities have their specific mechanisms to contribute to neuronal cell death and have been the subject of plethora of researches in the last decade.^3^

The amyloid hypothesis is recognized as the most plausible culprit for inducing AD and the therapeutic approaches were developed based on detachments of fibril monomers.^3^ However, failure of these approaches (for instance, the two bapineuzumab and solanezumab drugs, in phase III trials, were failed in 2012) raised the concerns whether the treatment approaches were designed in the right way (there is crucial questioning on the validity of Aβ hypothesis). Recently, the regulations of multivalent cations (i.e. Zn^2+^, Cu^2+^, Mn^≥2+^, Fe^≥2+^, and Co^≥2+^) in biological fluids have been received great attentions for AD community. The reason of this considerable attention is the fact that the unbalanced multivalent cations can act as linker among negatively charged Aβ monomers, and initiate the oligomerization process.^3^ Deep postmortem investigation, on the brain tissues of AD individuals, showed remarkable presence of multivalent cations in amyloid plaques.^3^

From the physiological points of view, similar to other tissues, the multivalent cations play crucial role in brain and are heterogeneously distributed with relatively high concentrations throughout the brain, due to the specific biological demands. As multivalent cations are inevitable parts of many proteins and enzymes possessing remarkable portions of human proteome, they may carry neuroprotective and neurotrophic properties in brain during ageing and restoration processes. However, studies have shown that these multivalent cations can strongly induce different aggregation pathways by utilizing the specific structural elements of human Aβ and potentially contribute to oxidative stress address the question whether their dyshomeostasis is the key cause for initiation of early molecular changes toward AD, repositioning the amyloid hypothesis in AD pathology. In this respect, I aimed to draw the attention of neurologist to a crucial role of unbalancing multivalent cations in biological fluid (i.e. blood plasma; as, very recently, we found that the serum concentrations of an array in such multivalent cations can be a fingerprint for identification of AD patients)^4^ due to the slight malfunction of the cationic regulating systems either in organic (e.g. liver and kidney) or molecular level (e.g. carrier proteins); more specifically, the diseases that have role in unbalancing of multivalent cations (e.g. hypertension, diabetes, insomnia, depression, and obesity) can be recognized as crucial silent initiator of AD. Thus, any insult in multivalent cations homeostatic machinery of the peripheral or central system at organ and molecular level may lead to the uncontrolled regulation and further loss of the original function in multivalent cations, which would be considered as the first molecular changes toward AD. As the first symptom of AD would be revealed 15-20 years after the initiation of the first molecular changing toward the AD,^3^ one can expect to have an effective therapy using intelligence multivalent cationic balance screening approaches for detection of AD at early stages.
